# The Quality of Educational Services from Students’ Viewpoint in Iran: A Systematic Review and Meta-analysis

**Published:** 2017-04

**Authors:** Ahmad MOOSAVI, Mohammad MOHSENI, Hajar ZIAIIFAR, Saber AZAMI-AGHDASH, Mahdi GHARASI MANSHADI, Aziz REZAPOUR

**Affiliations:** 1. Dept. of Health and Community Medicine, Dezful University of Medical Sciences, Dezful, Iran; 2. Health Services Management Research Center, Institute for Futures Studies in Health, Kerman University of Medical Sciences, Kerman, Iran; 3. Dept. of Health Management and Economics, School of Public Health, Tehran University of Medical Sciences, Tehran, Iran; 4. Iranian Center of Excellence in Health Management, Tabriz University of Medical Sciences, Tabriz, Iran; 5. Dept. of Health Services Administration, School of Public Health, Shahid Sadoughi University of Medical Sciences, Yazd, Iran; 6. Health Management and Economics Research Center, Iran University of Medical Sciences, Tehran, Iran; 7. Dept. of Health Economics, School of Health Management and Information Sciences, Iran University of Medical Sciences, Tehran, Iran

**Keywords:** Servqual, Services quality, Student, Meta-analysis, Iran

## Abstract

**Background:**

Students’ view is an important factor in assessing the quality of universities. Servqual pattern is regarded as the most prominent for services quality measurement. This study aimed to review systematically studies that investigated the quality of educational services.

**Methods:**

A systematic review and meta-analysis of studies evaluating students’ viewpoint about quality of educational services were conducted. Required data were collected from PubMed, Embase, Scopus, Science Direct, Google Scholar, SID, Magiran, and Iranmedex, without time restriction. Computer software CMA, ver. 2 was applied to estimate the total mean score of students’ perception and expectation of services quality and the gap between them.

**Results:**

The 18 eligible studies were entered into study. The studies were conducted between 2004 and 2014. Based on the random effect model, the total mean score of students’ perception, students’ expectation and the gap between them were estimated 2.92 (95% CI, 2.75 – 3.09), 4.18 (95% CI, 3.98 – 4.38), respectively and −1.30 (95% CI= −1.56, −1.04).

**Conclusion:**

The studied students’ expectation level is higher than the current quality of educational services. There is a tangible difference between their expectations and the current quality, which requires officials’ efforts to improve quality in all dimensions and effective steps can be taken towards improving the quality of educational services through appropriate training planning and training for empowering employees in colleges and universities.

## Introduction

Quality of services is an important factor for the growth, success and sustainability of an organization and is considered as a strategic, effective and overarching issue, which is put on the agenda of the management in most organizations (1). Given the importance of the issue, nowadays, there is great interest in the measurement of quality of service; however, quality is a subjective concept and difficult to measure and define (2, 3). In order to investigate the quality of services, customer expectations can be compared to his/her perceptions (what he/she has received). If from service recipient viewpoint, expectations are greater than perceptions, then the quality of services received is low and this can lead to his/her dissatisfaction (4). Meanwhile, the issue of perceptions and expectations when receiving services has a direct relationship with the level of understanding and awareness of the service recipients (5).

There are many patterns for services quality measurement and Servqual pattern is regarded as the most prominent of them. This pattern was offered in 1998 after conducting broad field studies in the field of quality of services. This pattern is a tool for measuring customer satisfaction with services that is based on the model of the gap between perceptions and expectations of services. This model is the most frequently used model for measuring services quality (6,7). Especially, studies show the use of this model in measuring the quality of educational services is also feasible (8). This tool was introduced by Parasuraman, which measures customers’ perceptions and expectations within five dimensions including Tangibility, Assurance, Empathy, Reliability and Responsiveness dimensions (9, 10).

Level of expectations of services quality in academic and educational environments due to the existence of special situation is at the highest level and consequently identifying the expectations and paying attention to the gap between the services offered and met expectations in such environments are matters of greater importance (11). In educational institutes, students are considered as main customers and in the meanwhile their viewpoints as the main and most important customers can play a significant role in improving services quality (4). In today’s world, students’ views about all aspects of education offered in educational institutions are investigated and considered as an important factor in assessing the quality of universities (12, 13).

Since one of the characteristics of quality in universities is to meet students’ expectations of educational services process, the quality of this process can be determined through by examining the gap between expectations and perceptions of students. A low gap between students’ expectations and perceptions indicates desired quality of educational services provided. Essential actions to narrow the gap include identifying students’ perceptions and expectations of the quality of educational services, determining strengths and weaknesses and, consequently adopting strategies for reducing the gap as well as satisfying students (14). In recent years, studies on quality of educational services and current gap in Iran have been conducted, however, in all reviewed studies there was a gap between students’ perceptions and expectations; this gap was negative (6,9 15–30). Furthermore, reviewing international studies indicated a negative gap between most of the dimensions (12, 31). The quality of services provided has been lower than students’ expectation. These studies alone do not provide a comprehensive view of decision-making and planning in order to improve the quality of services. Results of these studies should be gathered and analyzed systematically.

Thus, the current study aimed to review systematically studies, which used Servqual tools to investigate the quality of educational services from students’ viewpoint.

## Methods

A systematic review and meta-analysis of studies that evaluate students’ viewpoint about quality of educational services were conducted according to the Preferred Reporting Items for Systematic Reviews and Meta-Analyses (PRISMA) statement (32).

### Study identification

The required information was obtained from PubMed, Embase, Scopus, Science Direct, Google Scholar, SID, Magiran and Iranmedex using keywords: Servqual, gap, services quality, educational, educational services, student, Iran, and no restrictions were placed on study date. Reference management software (Endnote X5) was using to organize and assess the titles and abstracts, as well as to identify duplicate studies. Review articles on the quality of educational services and the reference lists of articles that satisfy the eligibility criteria were also hand-searched for additional articles.

### Study Selection

Two reviewers excluded articles with non-relevant titles and then, the abstracts and full texts of articles reviewed to include that matched the inclusion criteria. Disagreements were resolved by consensus with a third reviewer (A.R). Articles were included criteria: original articles, performed in educational setting, reported the mean score of student’ perception and expectation of educational services quality, published in English or Persian and conducted in Iran. Exclusion criteria were the proceedings papers, case reports, and interventional studies.

### Assessment of study quality

Two reviewers evaluated the articles based on the ‘Strengthening the Reporting of Observational Studies in Epidemiology’ (STROBE) checklist (33, 34).

### Data extraction

Two reviewers extracted data using a standard data collection form. Extraction table was designed that included the following items: author’s name, year of implementation, setting, sample and sample size, mean score of dimensions of educational services quality and significant factors were extracted (Table 1).

**Table 1:** Main characteristics of included studies

**Year of implementation**	**Sample size**	**Tangibles**			**Reliability**			**Responsiveness**			**Assurance**			**Empathy**		
		**M (P)**	**M (E)**	**Gap**	**M (P)**	**M (E)**	**Gap**	**M (P)**	**M (E)**	**Gap**	**M (P)**	**M (E)**	**Gap**	**M (P)**	**M (E)**	**Gap**
Abbasian et al: 2012	274	3.18	4.51	−1.33	3.41	4.56	−1.15	3.06	4.51	−1.45	3.41	4.55	−1.14	3.28	4.50	−1.22
Aghamolaei & Zare: 2007	300	3.10	3.94	−0.84	3.37	4.07	−0.71	2.78	3.92	−1.14	3.23	4.13	−0.89	3.07	4.03	−0.95
Kavosi et al: 2011	247	11.75	15.48	−3.72	11.78	18.29	−6.5	11.48	18.3	−6.81	11.97	18.57	−6.6	11.25	18.37	−7.11
Rasoolabady et al: 2011	198	3.3	4.49	−1.19	4.21	4.44	−0.23	2.88	4.32	−1.44	3.01	4.36	−1.35	3.43	4.38	−0.95
Tofighi et al: 2010	170	2.14	3.53	−1.11	2.30	3.61	−1.31	2.18	3.60	−1.42	2.25	3.65	−1.40	2.03	3.61	−1.57
Enayati et al: 2010	373	2.71	4.75	−2.03	2.54	4.73	−2.17	2.63	4.76	−2.13	2.44	4.72	−2.27	2.40	4.65	−2.25
Ayatollahi et al: 2010	68	3.2			3.42			3.02			3.28			3.27		
Bahreini et al:2009	220	2.65	3.35	−0.70	3.15	3.25	−0.10	2.28	2.83	−0.55	3.13	3.26	−0.13	2.86	3.24	−0.38
Mohebbifar et al, 2011	256	2.90	4.65	−1.74	3.16	4.57	−1.41	2.88	4.84	−1.96	3.36	4.60	−1.24	3.06	4.53	−1.47
Bahadori et al, 2012	383	2.38	4.08	−1.69	2.75	4.08	−1.33	2.22	3.99	−1.76	2.38	4.13	−1.75	2.48	4.02	−1.54
Yazdi Feyzabadi et al, 2011	303	3.25	4.11	−0.86	3.33	4.24	−0.91	3.04	4.16	−1.11	3.28	4.12	−0.84	3.24	4.24	−1.00
Gholami, A et al: 2014	198	3.07	4.37	−1.31	3.38	4.40	−1.02	3.04	4.34	−1.30	3.21	4.47	−1.26	3.16	4.49	−1.33
Haresabadi M et al: 2011	175	2.3	4.2	−1.9	2.7	4.3	−1.6	2.4	4.2	−1.8	2	4.2	−2.2	2.4	4.2	−1.8
Bakhshi, H et al: 2008	310	2.79	4.6	−1.8	3.3	4.6	−1.4	2.83	4.49	−1.6	2.98	4.5	−1.5	3.60	4.49	−1.4
Goharinezhad, S et al: ---	*97*	3.15	4.42	−1.28	3.43	4.69	−1.26	2.96	4.62	−1.66	3.04	4.61	−1.56	3.22	4.47	−1.23
Arbouni, F: 2007	362	2.21	3.73	−1.52	3.77	2.31	−1.46	2.13	3.75	−1.62	2.24	3.78	−1.54	2.08	3.75	−1.67
Nabilou, B and Khorasani−Zavareh, D: 2007−8	173	3.49	4.38	−0.89	3.37	4.20	−0.83	3.07	4.16	1.09	3.52	4.48	−0.96	2.99	4.1	−1.11
Nabilou, B and Khorasani-Zavareh, D: 2007–8	173	3.02	4.11	−1.09	2.77	4.27	−1.5	2.58	4.06	−1.48	2.52	4.1	−1.58	2.33	3.8	1.47
Miri, S et al: 2008	485	2.98			3.42			2.96			3.26			3.33		
Kebriaei, A and Roudbari M: 2004	386	2.72	4.03	−1.31	3.20	4.30	−1.10	2.38	4.11	−1.73	2.75	4.29	−1.54	2.81	4.36	−1.55

P: Perception/ E: Expectation

### Data analysis

To estimate the overall mean score of educational services quality computer software CMA 2 (Comprehensive Meta-analysis) (Englewood, NJ, USA) was used. Forest plots with 95% confidence interval were used. The Cochrane Q statistic was calculated to assess heterogeneity of results in different studies and I^2^ was used to quantify the magnitude of between study heterogeneity (Q statistic *P-* value<0.05 or I^2^>50%). Funnel plot was applied to evaluate the possibility of publication bias and Excel 2010 was used to draw graphs.

## Results

**Fig. 1:**
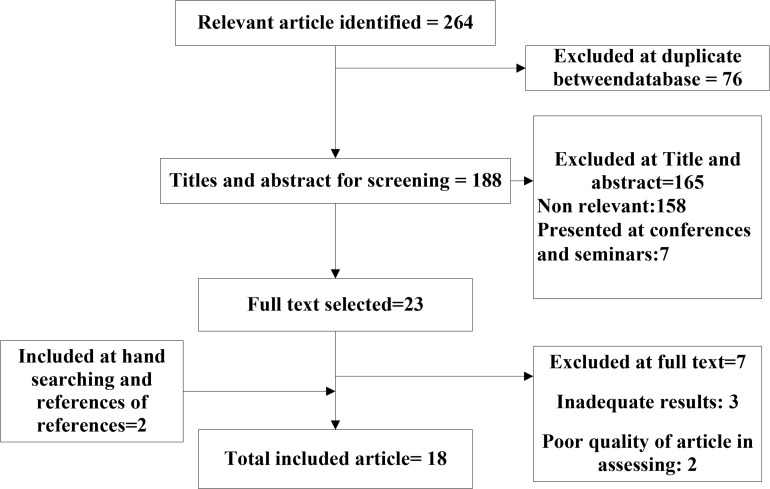
Flow diagram for study selection

Results in relation to 4904 people mentioned in 18 articles were considered. Fig. 1 shows the flowchart for the identification of studies. The studies were conducted between 2004 and 2014.

The total mean score of students’ perception, students’ expectation and gap between them was estimated to be 2.92, 4.18 and −1.30, respectively.

The highest and lowest mean scores for students’ perception of services quality were 3.37 and 2.18, respectively. However, the highest and lowest mean scores for expectation of services quality were 4.72 and 3.15, respectively. In relation to gap, the highest and lowest levels were −2.17 and 0.37, respectively. The total mean score of students’ perception based on the random effect model was estimated to be 2.92 (95% CI, 2.75–3.09).

**Fig. 2:**
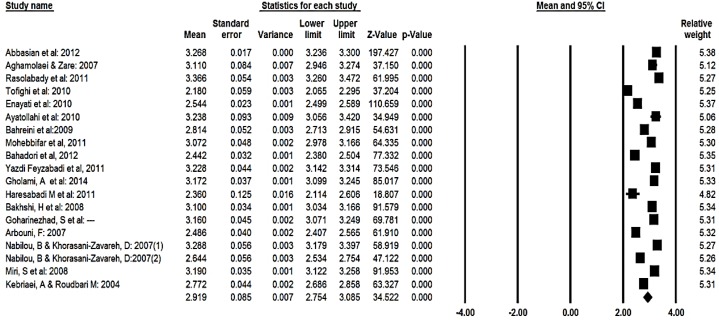
The total means score of students’ perception

**Fig. 3:**
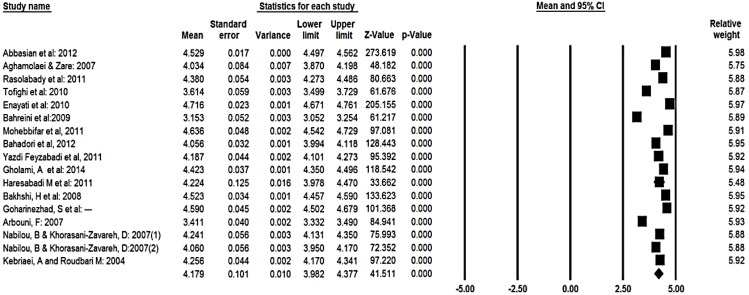
The total means score of students’ expectation

**Fig. 4:**
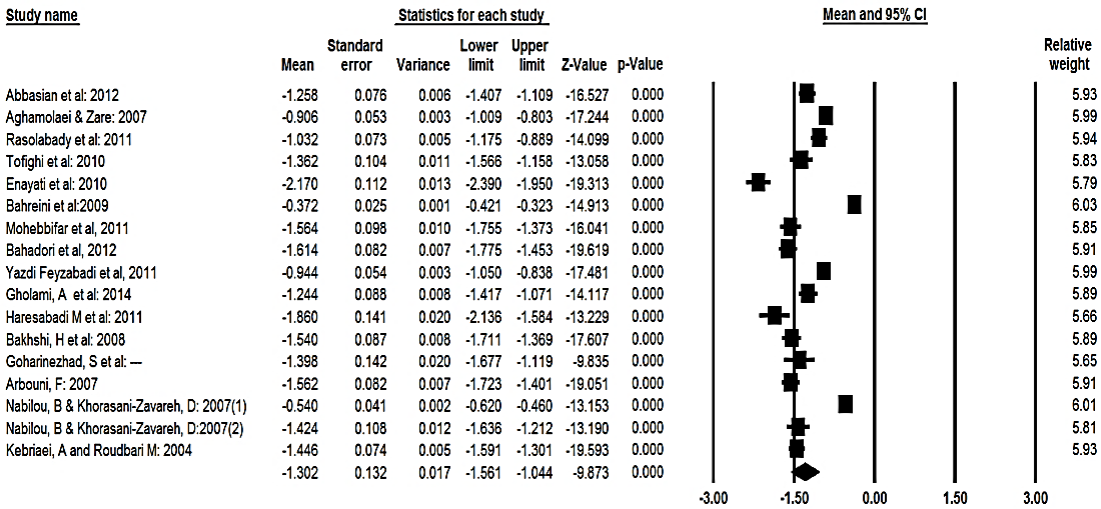
The total means score of Gap between expectations and perceptions

Ninety-five percent CI for the mean score was drawn for each study in the horizontal line format (Q=740, df=18, *P*<0.001, I^2^=97.5%) (Fig. 2). The total mean score of students’ expectation based on the random effect model was estimated to be 4.18 (95% CI, 3.98 – 4.38). For the mean score 95% CI was drawn for each study in the horizontal line format (Q=1860, df=16, *P*<0.001, I^2^=99.4%) (Fig. 3). Moreover, the total mean score of gap between perception and expectation based on the random effect model was estimated to be −1.30 (95% CI=−1.56, −1.04). For the mean score 95% CI was drawn for each study in the horizontal line format (Q=1082, df=16, *P*<0.001 I^2^=98.5%) (Fig. 4). To evaluate the publication bias, funnel plot was applied. Result of this funnel plot show there was possibility publication bias among studies.

**Fig. 5:**
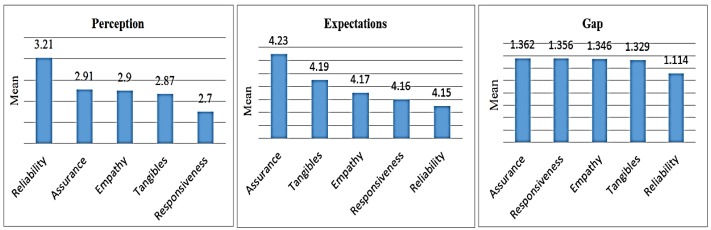
The mean score of students’ perception, expectations and gap of services quality

Among total mean scores of the five dimensions of perception of services quality, in all studies, reliability dimension had the highest mean score (3.21) while the responsiveness dimension had the lowest one (2.70). This means that the respondents assigned the lowest point to the responsiveness dimension and, there was a lower perception of this dimension in educational services among them. Among total mean scores of the five dimensions of expectation of services quality, in all studies, assurance dimension had the highest mean score (4.23) while the reliability dimension had the lowest one (4.15). This means that the respondents’ highest expectation of services quality was related to the assurance dimension. And among total mean scores of the five dimensions of gap between perception and expectation of services quality, in all studies, the highest gap was related to assurance dimension (−1.36) while the lowest gap was related to the reliability dimension (−1.11) (Fig. 5).

## Discussion

Students’ perception, students’ expectation and the gap between them were estimated respectively 2.92 (95% CI, 2.75 – 3.09), 4.18 (95% CI, 3.98 – 4.38) and −1.30 (95% CI= −1.56, −1.04). The studied students’ expectation level is higher than the current quality of educational services and there is a tangible difference between their expectations and the current quality, which requires officials’ efforts to improve quality in all dimensions.

The quality gap was negative in each of the five dimensions in the healthcare centers. A negative quality gap indicates that there is a gap between customers’ expectations and perceptions of service and their expectations have not been met adequately. The results of the studies on services quality in hospitals and healthcare centers also show a negative gap between patients’ and service recipients’ expectations and perceptions of the quality of services provided in the studied hospitals (9, 35–43). The negative gap between expectations and perceptions can be resulted from various issues such as limited resources and equipment, lack of proper planning, lack of officials’ attention to service recipients’ expectations and wants, high level of expectation among people and so on (36). Quality improvement programs can greatly reduce this gap. Limited recourses is one of the main barriers of the implementation of services quality improvement programs (44), therefore, conducting research on quality of service, in addition to the identification of problems, will mobilize limited resources to the areas to be able them to meet service recipients’ expectations of services quality as much as possible.

In this study, among the five dimensions of perception of service quality, while the reliability dimension (3.21) has the highest score, the responsiveness dimension has the lowest one (2.70). The responsiveness dimension is as the same as willingness to cooperate and assist the customer (45, 46) students’ perception of the status of the responsiveness dimension was lower than the expected level. In order to improve the quality of educational services, all the dimensions, especially the responsiveness dimension, should be taken into consideration. In relation to the responsiveness dimension, factors such as the lack of easy access to management, lack of adequate access to professors and ignorance of students’ views in developing educational programs, can play a great role in students dissatisfaction. The issue of responsiveness has always had some difficulties. In the US, the studied students mentioned the existence of a gap between perception and expectation in the responsiveness dimension as one of the main factors of their dissatisfaction (47).

Among the five dimensions of expectation of quality, while the assurance dimension had the highest score (4.23), the reliability dimension had the lowest one (4.15). Reliability means that services are provided in a reliable and trustworthy manner (45,46). Holding briefing sessions for staff, continuous in-service training for staff, training of the importance of service recipients’ satisfaction and the important role of customer in organizations can partially meet expectations of recipients of services and reduce the gap between perception and expectation.

In relation to the five dimensions of the gap between perception and expectation of services quality, the highest gap was related to the assurance dimension (−1.36) and the lowest gap was associated to the reliability dimension (−1.11). The highest gap was related to the empathy dimension (36). In addition, the highest gaps were related to the empathy and reliability dimensions, respectively (16, 40). The highest gap was related to the responsiveness dimension (46, 48). In addition, the lowest gap was seen in the tangibles dimension (9, 46). The lowest gap was associated to the reliability dimension that was consistent with the results of the current study (18, 36, 49, 50). The existence of defect and gap in one dimension has a negative effect on the other dimensions. From service recipients’ viewpoint, other dimensions of service quality are underestimated. If an educational system is in a desired situation in terms of quality, then it can perform their tasks properly. The necessity of finding ways to increase education quality seems necessary (28). The identification of customers’ wants and perception of quality of services is a major step to reduce the current gap and difference between expectations and perceptions of service recipients. In this case, through identifying current differences, not only resources allocation will be facilitated, however, there will be a basis for improving the quality of services provided.

## Conclusion

Standards related to continuous quality improvement can be used to improve and promote the quality. Based on dimensions of quality that have serious problems, management of academic centers should prioritize and plan properly. Using appropriate tools for organization within new management of organizations can help the achievement of organizational goals in order to improve quality. In addition, using views of customers, which are students, re-engineering of processes and taking more advantage of quality improvement techniques should be considered. Finally, using appropriate training planning in the field of specialized knowledge of professors as well as training to improve the ability of the staff in colleges and universities effective steps can be taken to improve the quality of educational services in universities.

## Ethical considerations

Ethical issues (Including plagiarism, informed consent, misconduct, data fabrication and/or falsification, double publication and/or submission, redundancy, etc.) have been completely observed by the authors.
